# Supplementary material for: Development of a bioassay method to test activity of cry insecticidal proteins against Diatraea spp. (Lepidoptera: Crambidae) sugarcane stem borers
Source: PLoS One. 2023 Oct 18;18(10):e0292992. doi: 10.1371/journal.pone.0292992 (PMC10584178; doi:10.1371/journal.pone.0292992)
Supplement: Attachment — Submitted filename: PONE D-23-19646_Reviewer1.pdf [file pone.0292992.s003.pdf]

PONE-D-23-19646

This is an interesting adaptation of a protocol to assess the susceptibility of sugarcane borers. If thoroughly explained, it can become a standard protocol for others to follow. Some of the necessary additions and clarifications are:

Line 103: 'Corn cobs' requires a full description of the maize cultivar, its agronomic management, the test to verify that the cultivar did not express any Bt proteins, when it was harvested and how, etc.

Lines 135-137: …24.1 μg/cm2 of Cry1Ac…was selected in assessing toxicity based on previously reported LC_{50} values for Cry1Ac from diet surface contamination bioassays with *D. saccharalis* and *D. flavipennella.* According to Lemes et al. [20], the LC_{50} for the latter is LC_{50} = 495ng/cm2 Why the difference?

Lines 275-277: The lack of available artificial diets supporting larvae growth has prevented testing susceptibility to these insecticidal proteins in other relevant *Diatraea* species. According to the text before and after this statement, THERE IS artificial diet for *D. saccharalis*, and *D. flavipennella*. Perhaps a little bit of effort with successful artificial diets for these two pests can be adapted for the other two. To propose your protocol as a 'standard', a full description of the maize cultivar, its growth and other parameters need to be provided. Has this protocol tested with other cultivars and on different leaves?

Lines 297-298: …meaning that more than double of the protein is required for coating the tissue (i.e., 24.1 μg/cm2) and effectively expose the insects... I would be important to note that production of Cry1Ac is not easy, neither cheap. Of greater value could be the cost comparison between this method and the use of artificial diet.

In the conclusions section I could not find anything different of what was described in the abstract or complemented in the text. Is it necessary?

Minor comments.

Line 11: I believe it should be CIAT

Line 30: Because there is no described artificial diet and availability of most purified Bt toxins,…

Line 39: Remove species evaluated

Line 47 mentions: cause significant yield losses. Provide the range, rather than requiring the reader to search for references 3 and 4.

Lines 51 and 52: with production losses (Provide the range) depending on the crop stage and damage caused (Describe)

Line 56: infection by *Coletotrichum falcatum*, a fungus that results~~ing~~

Line 58: …as a reduction in both total biomass production and sucrose content. Provide ranges.

Line 75: application Suggestions; alternative / option

Line 78: , or by direct observation of their efficacy in plants in the field (Dively et al. 2023 [https://www.mdpi.com/2075-4450/14/7/577], and previous publications)

Line 97: Entomology Laboratory does not require capital letters. It is only ‘a room’.

Line 103: Fix F_1

Lines 104-105: if the thickness of the cob has a variation of 20%, then the term ‘uniform’ disk is not correct. Just saying.

Line 126: on ~~four *Diatraea* species,~~ *Diatraea*~~.~~ *saccharalis*

Lines 127-128: By the time maize is ready to provide corn discs 75 days after planting, a plant may have at least 12 leaves at different stages of development and thickness. Provide information on the precise development stage of maize using the ISU scale (https://store.extension.iastate.edu/product/Corn-Growth-and-Development), and describe which maize leaf(ves) was used in the bioassays.

Line 133: was …water and detergent alone… only used in the control? If so, specify / justify why.

Line 141: Describe how ~~M~~mortality was scored 7 days after infestation

Lines 153-155: Is it necessary to describe this simple formula? The same one described in line 188.

Line 196: It is described that “The 16 wells within each of the eight replicates were considered subsamples” Is this a replication, made only once with one corn borer generation? Specify.

Line 225: “almost all larvae were stunted” requires precision.
